# Supplementary material for: The Misconception of Antibiotic Equal to an Anti-Inflammatory Drug Promoting Antibiotic Misuse among Chinese University Students
Source: Int J Environ Res Public Health. 2019 Jan 25;16(3):335. doi: 10.3390/ijerph16030335 (PMC6388115; doi:10.3390/ijerph16030335)
Supplement: Supplementary File 1 [file ijerph-16-00335-s001.pdf]

Questionnaire of antibiotic use among Chinese university students

Part 1: The General Information

1. Your university:

   ☐Zhejiang University; ☐Wuhan University; ☐Tianjin University;

   ☐Jilin University; ☐Lanzhou University; ☐Guizhou University;

2. Your date of birth： Year_______ Month________

3. Your gender：

   ☐male ☐female

4. Your education level：

   ☐undergraduate student; ☐master student; ☐Ph.D. student

5. Your grade:_____________

6. Your major：

   ☐science ☐social science and humanity ☐medicine

7. Your father's education level:

   ☐illiteracy and primary school ☐middle school ☐high school

   ☐junior college ☐undergraduate and higher

8. Your mother's education level:

   ☐illiteracy and primary school ☐middle school ☐high school

   ☐junior college ☐undergraduate and higher

9. Does your father have a medical background?

   ☐yes ☐no

10. Does your mother have a medical background?

    ☐yes ☐no

11. Your household income per month (RMB) in the past year?

    ☐<3,000 ☐3,001-10,000 ☐10,001-20,000 ☐>20,000

12. Pelase tell us the province of your hometown: ___________

13. Please tell us your residence in your hometown: ☐rural ☐urban

Part 2: Antibiotic Use Misconception

14. Antibiotic is a *Xiaoyanyao.*

    ☐yes ☐no ☐do not know

Part 3: Antibiotic Use Behaviors

Please read the following messages before you start the next part of the survey.
Antibiotics are not anti-inflammatory drugs. Common-used antibiotics include:

- Penicillin (amoxicillin, etc.)
- Cephalosporin (cefaclor, ceftriaxone sodium, etc.)
- Quinolones (norfloxacin, ofloxacin, etc.)
- Macrolides (azithromycin and roxithromycin etc.)
- Sulfa

15. Did you get sick in the last month?

    ☐yes ☐no（skip to No.22）

16. If yes, which conditions did you get? (you can choose more than one answer)

    ☐sore throat ☐common cold (cough, runny nose) ☐headache ☐otitis media

    ☐flu ☐diarrhea ☐others（please specify）_______________

17. What treatment did you get？

    ☐pure self-treatment without advice from a doctor, nurse, or dentist

    ☐go to see a doctor, nurse, or dentist (skip to No.22)

    ☐do nothing (skip to No.22)

18. If it is pure self-treatment, did you use antibiotics?

    ☐yes ☐no (skip to No.22)

19. If yes, please tell us the name of antibiotics ___________________

20. If you went to see a doctor, nurse, or dentist, which type of health facilities did you choose?

    ☐private clinic ☐community health service center/station

    ☐county level hospital ☐county level above hospital

21. If you went to see a doctor, nurse, or dentist, did you get antibiotics?

    ☐yes, please tell us the antibiotic name______________

    ☐no (skip to No.22)

22. Did you take a request to obtain antibiotics (or get infusion) from doctors in the past year?

    ☐yes ☐no

23. Did you take antibiotics to prevent diseases (such as colds, flu etc.) without advice from a doctor, nurse, or dentist in the past year?

    ☐yes ☐no
